# Voluntary Medical Male Circumcision (VMMC) in Tanzania and Zimbabwe: Service Delivery Intensity and Modality and Their Influence on the Age of Clients

**DOI:** 10.1371/journal.pone.0083642

**Published:** 2014-05-06

**Authors:** Tigistu Adamu Ashengo, Karin Hatzold, Hally Mahler, Amelia Rock, Natasha Kanagat, Sophia Magalona, Kelly Curran, Alice Christensen, Delivette Castor, Owen Mugurungi, Roy Dhlamini, Sinokuthemba Xaba, Emmanuel Njeuhmeli

**Affiliations:** 1 Maternal and Child Health Integrated Program (MCHIP), Washington, District of Columbia, United States of America; 2 Johns Hopkins University, Bloomberg School of Public Health, Baltimore, Maryland, United States of America; 3 Population Services International (PSI), Harare, Zimbabwe; 4 MCHIP Tanzania, Dar es Salaam, Tanzania; 5 JSI Research & Training Institute, Inc., Boston, Massachusetts, United States of America; 6 United States Agency for International Development, Washington, District of Columbia, United States of America; 7 Ministry of Health and Child Welfare, Harare, Zimbabwe; World Health Organization, Switzerland

## Abstract

**Background:**

Scaling up voluntary medical male circumcision (VMMC) to 80% of men aged 15–49 within five years could avert 3.4 million new HIV infections in Eastern and Southern Africa by 2025. Since 2009, Tanzania and Zimbabwe have rapidly expanded VMMC services through different delivery (fixed, outreach or mobile) and intensity (routine services, campaign) models. This review describes the modality and intensity of VMMC services and its influence on the number and age of clients.

**Methods and Findings:**

Program reviews were conducted using data from implementing partners in Tanzania (MCHIP) and Zimbabwe (PSI). Key informant interviews (N = 13 Tanzania; N = 8 Zimbabwe) were conducted; transcripts were analyzed using Nvivo. Routine VMMC service data for May 2009–December 2012 were analyzed and presented in frequency tables. A descriptive analysis and association was performed using the z-ratio for the significance of the difference. Key informants in both Tanzania and Zimbabwe believe VMMC scale-up can be achieved by using a mix of service delivery modality and intensity approaches. In Tanzania, the majority of clients served during campaigns (59%) were aged 10–14 years while the majority during routine service delivery (64%) were above 15 (p<0.0001). In Zimbabwe, significantly more VMMCs were done during campaigns (64%) than during routine service delivery (36%) (p<0.00001); the difference in the age of clients accessing services in campaign versus non-campaign settings was significant for age groups 10–24 (p<0.05), but not for older groups.

**Conclusions:**

In Tanzania and Zimbabwe, service delivery modalities and intensities affect client profiles in conjunction with other contextual factors such as implementing campaigns during school holidays in Zimbabwe and cultural preference for circumcision at a young age in Tanzania. Formative research needs to be an integral part of VMMC programs to guide the design of service delivery modalities in the face of, or lack of, strong social norms.

## Introduction

More than 40 observational studies and three randomized controlled trials have demonstrated that voluntary medical male circumcision (VMMC) reduces HIV acquisition among heterosexual men by approximately 60% [1], [2], [3]. Post-trial follow-up maintained for up to 4.9 years indicates that this protective effect was durable and even increased over time [4]. Data from a 2011 South African study by Auvert et al. found a 76% reduction in new HIV infections among circumcised men [5]. Consequently, the World Health Organization (WHO) and the Joint United Nations Programme on HIV/AIDS (UNAIDS) recommended that VMMC should be part of comprehensive HIV prevention programming in regions with relatively high HIV prevalence and lower levels of male circumcision. Mathematical modeling suggests that approximately 3.36 million new HIV infections and 386,000 AIDS deaths would be averted through 2025 if 80% coverage with VMMC is achieved within five years in 13 Eastern and Southern Africa priority countries [6]. In order to achieve this level of coverage, an estimated 20.3 million VMMC procedures among men age 15 to 49 would need to be performed; an estimated 3.2 million of which had been conducted by December 2012 [7]. Despite several initiatives to increase VMMC coverage in these 13 settings, the progress has been modest. While there are still service supply constraints in some areas, demand creation and uptake of services is a well-recognized barrier to achieving VMMC targets.

In order to reach 20.3 million with VMMC service, these barriers must be understood, prioritized and addressed as part of an efficient and effective VMMC program. VMMC programs in many countries have adopted a model of service provision whereby services are provided not only in fixed health facilities, but also in outreach sites and mobile units. In addition to diversifying the type of service outlets, attempts to increase circumcision through periods of high intensity and campaign approaches have also been utilized in many countries, including Tanzania and Zimbabwe. In this paper, we explored the role of these different service delivery modalities and intensity approaches in improving performance as well as attracting or preventing men of certain age from seeking and accessing VMMC services. The review specifically looks at the cases of Tanzania and Zimbabwe.

Both Tanzania and Zimbabwe are among the 13 priority countries where WHO and UNAIDS recommended that VMMC be implemented as a part of their HIV prevention programs [8].

**In Tanzania**, only one region, Iringa (which was recently divided into two administrative regional units, Iringa and Njombe), will be used for illustration and discussion in this paper. A national strategy launched in 2010 [9] promoted scale-up of VMMC as a prevention intervention per WHO's and UNAIDS' recommendations. To scale up VMMC, Tanzania has embraced task shifting and task sharing. (Task shifting is a process of delegation: tasks are moved, where appropriate, from specialized to less specialized health care workers. Task sharing is needs-based, and uses a team approach; for VMMC it is the use of lesser-trained cadres to perform particular steps in male circumcision surgery.) By reorganizing the workforce in this way, task shifting presented a viable solution for improving coverage by more efficiently using human resources already available and quickly increasing capacity while training and retention programs are expanded [10]. However, despite the use of task shifting/sharing approaches, progress was still modest and other ways of improving coverage were sought, including utilizing outreach services and limited-time campaigns for VMMC services.

**Figure 1:**
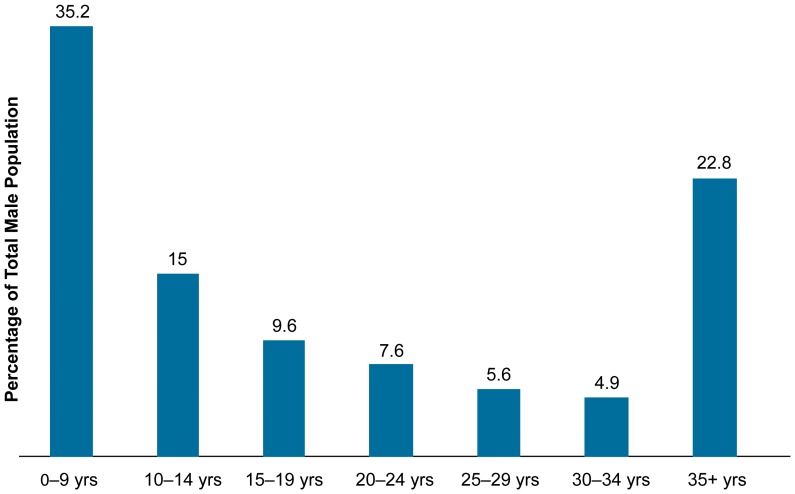
Percentage Age Distribution of Total Male Population in Tanzania (all regions).

The national VMMC strategy targets males between the ages of 10–34 years, who comprise 42.7% of male population (Figure 1), in regions with high HIV prevalence and low male circumcision prevalence (Iringa, Njombe, Mbeya, Rukwa, Katavi, Shinyanga, Geita, Mara, Mwanza, Tabora, Simiyu, Kagera: 12 of the 30 Tanzanian regions). However, strong cultural norms favor circumcision before or during puberty [11].

Since 2009, when the program began with a small pilot, the vast majority (82%) of VMMC clients in Iringa and Njombe were adolescents between the ages of 10–19. Beginning in 2011, the program revamped its demand creation strategy to tailor communication messages to men age 25 and over. However, adolescents continue to comprise the majority of VMMC clients. While it is beneficial to provide services to adolescent boys and younger men, the large number of adolescent clients might further hinder the adoption of the procedure by older men, by reinforcing the existing cultural norm that VMMC is best done before or during puberty [11]. The manner in which male circumcision is introduced will have a direct impact on its future adoption. How an issue such as male circumcision is introduced will influence the attitudes of policy makers and the public about the procedure and thereby facilitate or impede utilization [12].

**Figure 2:**
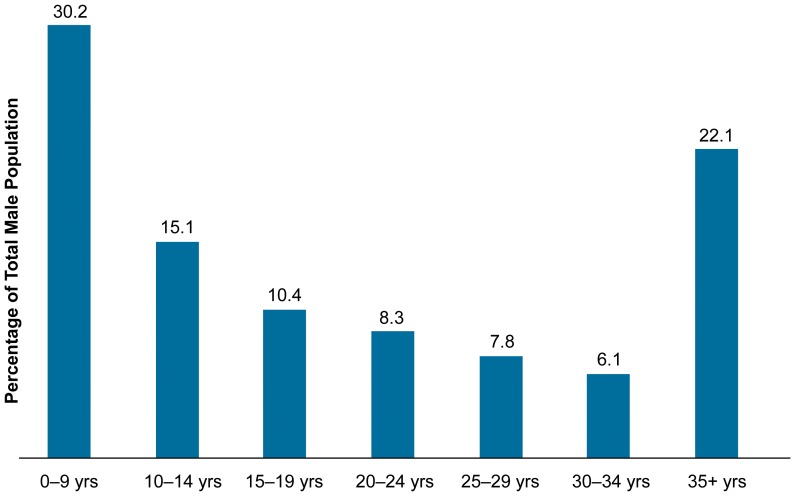
Percentage Age Distribution of Total Male Population, Zimbabwe.

**In Zimbabwe**, according to the 2010–11 Zimbabwe Demographic and Health Survey, HIV prevalence among men age 15–49 is 15.2 percent (54.9% of the male population—Figure 2) [13]. The national HIV response is coordinated by the National AIDS Council through the Zimbabwe National HIV and AIDS Strategic Plan. National plans have stressed evidence and results-based strategies, with HIV prevention the cornerstone of the response [14]. In a large stakeholders meeting in 2007, the Zimbabwean Government adopted VMMC as a priority HIV prevention strategy and created a steering committee and three technical working groups to initiate a pilot program in May 2009.

In December 2009, the Ministry of Health and Child Welfare (MOHCW) Zimbabwe launched the National Male Circumcision Policy for HIV Prevention as part of an integrated response and the VMMC strategy, which aims to reach 80 percent of males aged 13–29 (1.3 million) between 2011 and 2015 [14].

Zimbabwe was one of the first countries adopting most of the elements to improve efficiency of VMMC services according to WHO guidelines, which were used in the national scale-up process [15]. Specifically, the program uses the forceps-guided method as a standard surgical procedure; all sites use several surgical bays and rotate providers between open operating rooms with curtains or screens separating individual bays. Doctors and nurses share tasks during the surgical procedure [16]. Sites use bundled, pre-sterilized and pre-packed disposable instrument sets and supplies throughout the program. Both fixed and outreach sites use electrocautery/diathermy to achieve hemostasis, further increasing efficiency and timing of the surgical procedure. The program uses a mix of fixed sites, which are either co-located within existing health care facilities at central, provincial and district levels, or stand-alone sites. Outreach services, whereby mobile VMMC units visit lower level health care facilities and clinics on a regular basis, were scaled up substantially during 2011 and 2012 and represents the main model of VMMC service delivery in Zimbabwe. By December 2012, the program had 12 fixed sites, 32 outreach sites and 27 mobile teams across all 10 provinces and offered VMMC through routine and campaign service delivery modalities [16], [17].

While supply-side efficiency has been high since the beginning of the program, demand creation has been challenging in Zimbabwe, a country where male circumcision is traditionally only practiced by a minority of ethnic groups. To increase uptake of VMMC, since 2011 the program introduced campaigns mainly during school holidays in April/May, August/September and November/December, each lasting four to six weeks. During campaigns, additional service delivery sites are identified, including health clinics at workplaces in mining and commercial farming areas as well as educational institutions, schools and universities. Demand creation is intensified during campaigns using a combination of mass media and interpersonal communications. Close collaboration between the MOHCW and the Ministry of Education allow for mobilization in schools and tertiary institutions involving head masters, teachers, youth VMMC champions and parents.

## Methods

VMMC service delivery data from national and two implementing partners were abstracted and analyzed. Routine VMMC service data for the period May 2009–December 2012 were analyzed with regard to the number of VMMC clients reached and the age distribution of clients during periods of campaign and routine service delivery at fixed, outreach and mobile sites. A total of 21 key informants (eight in Zimbabwe, 13 in Tanzania) were interviewed. Respondents in Tanzania included Ministry of Health and Social Welfare (MOHSW) clinicians, medical officers and program staff; Maternal and Child Health Integrated Program (MCHIP) staff; and a United States Agency for International Development (USAID) HIV prevention advisor. In Zimbabwe, respondents comprised MOHCW and Population Services International (PSI) VMMC program staff, University of Zimbabwe faculty, HIV counseling and testing program staff, service providers and a member of parliament. Key informant interview transcripts were analyzed using NVivo qualitative software, service statistics were summarized and presented in simple frequency tables, a descriptive analysis was performed and association between age and type of service delivery modality (routine and campaign- Table 1) was calculated using the z-ratio for the significance of the difference between two independent proportions.

**Table 1:** Site Options and Service Delivery Modalities.

PEPFAR's *Best Practices for Voluntary Medical Male Circumcision Site Operations* * provides the following definitions for site options and service delivery modalities for countries implementing VMMC programs
**VMMC Site Options:**
**Fixed sites** are permanent structures—often located near or within existing health care facilities—that offer VMMC services on a continuous basis. Fixed sites may serve as a hub for multiple mobile units.
**Mobile sites** are usually temporary structures, often tents and prefabricated structures, which can be used for HIV testing and counseling services at the VMMC site, performing follow-up visits or group education.
**Outreach sites** can be permanent structures (e.g., primary clinics or schools) modified for VMMC service purposes, or temporary structures to increase available space so more clients can receive VMMC services.
**Service Delivery Modality:**
**Routine** service delivery ensures the availability of VMMC services at existing health care facilities year round. Although space may be dedicated solely to VMMC services within a facility, the services are integrated with overall facility services and offered consistently throughout the year.
**Campaign** service delivery provides VMMC services in high volume for short periods of time. With campaign service delivery, commodities and human resources are dedicated for the duration of the campaigns. Demand creation and community sensitization are crucial components to ensure a high volume of demand for VMMC services during the campaign period.

*PEPFAR's Best Practices for Voluntary Medical Male Circumcision Site Operations: A service guide for site operations, 2013. Available: http://www.usaid.gov/sites/default/files/documents/1864/pepfar_best_practice_for_vmmc_site_operations.pdf.

The Johns Hopkins School of Public Health and John Snow Incorporated Institutional Review Boards (IRBs) reviewed the request for secondary data analysis. The Johns Hopkins School of Public Health IRB has determined that the secondary data analysis activity meets the criteria for Exemption under 45 CFR 46.101(b), Category (4) IRB No: 00004168 and provided an exemption. The John Snow, Inc. IRB also deemed this study exempt from oversight. All clients accessing VMMC services in the two countries have their information recorded and kept at health facilities as per the rules and guidelines of the governments of Tanzania and Zimbabwe. For all the interviews as part of this analysis, the interviewer obtained written consent from all interviewees before conducting interviews.

## Results

### Qualitative

**Tanzania**, “*Benefit of an outreach is you can reach people who cannot come to a fixed site. Communities are very scattered, very far*,” (key informant). Respondents reported that VMMC service should be provided through fixed and outreach sites to improve access. And they believe mobile sites are preferred to provide counseling and testing and other services if facilities are low on space. According to the informants, the decision to offer services at fixed sites during a routine or campaign schedule depends on whether the catchment area around the fixed sites had been exhausted through previous campaigns and routine service delivery. The fact that service is provided within the existing health services infrastructure, the service providers are from the public health sector and services are provided free of charge attract younger clients to easily access services. Respondents believe that older clients may prefer services that do not accept younger clients and that provide services after work hours or on weekends. Respondents also indicated that preference to circumcise at a younger age is very common and that it is difficult to get older client to come forward for services.

**Zimbabwe** also provides VMMC services through fixed, mobile and outreach sites. Respondents believe that the main mode of service delivery is and should be outreach because the majority of the population cannot access fixed sites. Health providers also agree with the expansion of outreach services but prefer surgical procedures to be done in facilities for infection control and therefore are only comfortable with using tents for counseling when facilities are low on space. Neither the organization of services as integrated or co-located at public health facilities nor the provision of services free of charge appear to effect the age of clients accessing services according to the respondents. Key informants also emphasized the importance of working with communities and understanding and addressing their concerns. Community partnerships were discussed as being crucial to VMMC program success. *“Talk with and not to communities you want to circumcise,” professor, University of Zimbabwe*.

### Quantitative

**Figure 3:**
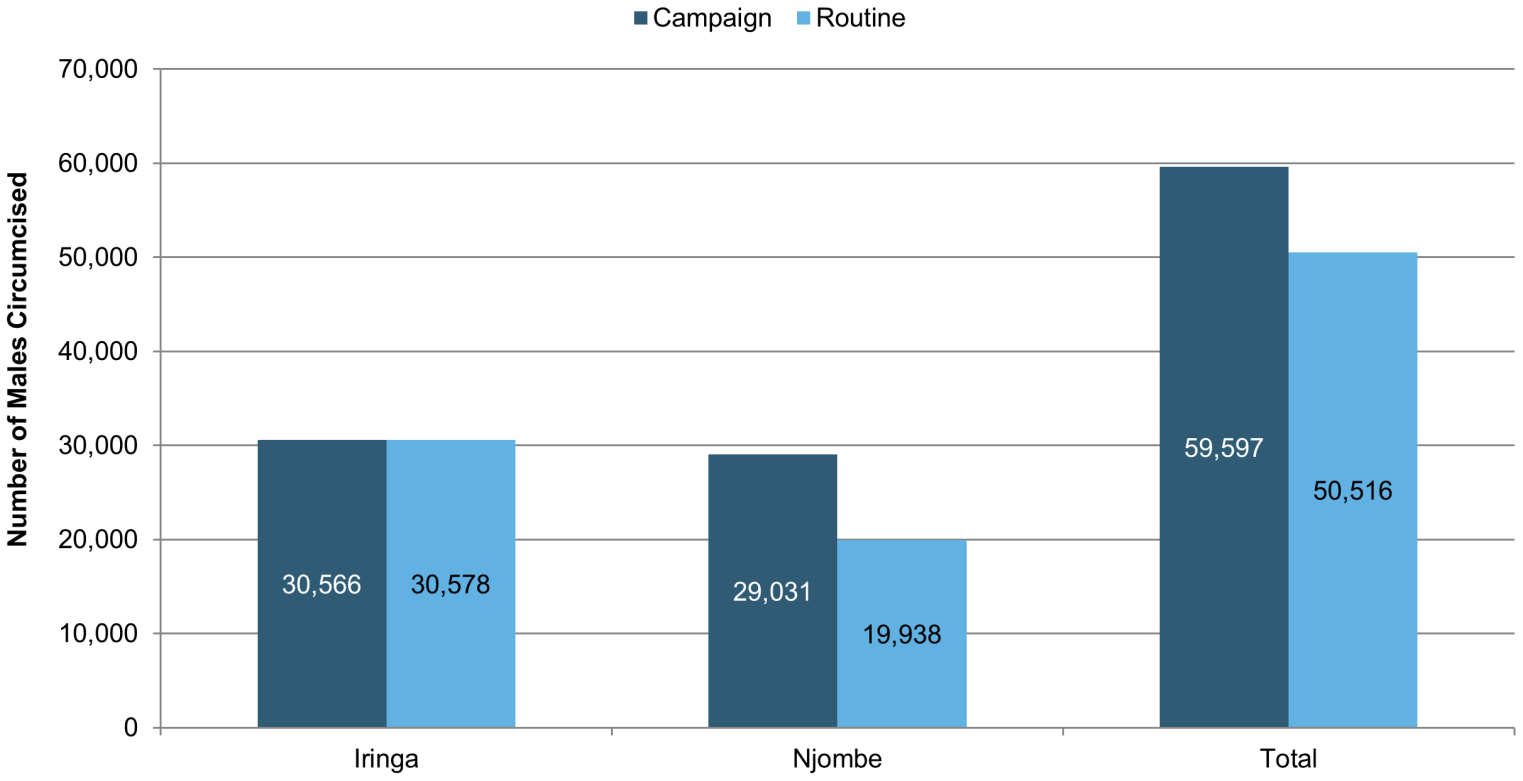
Total number of VMMC procedures in Iringa and Njombe regions, Tanzania, October 2009 to December 2012.

**In Tanzania**, 110,113 males were circumcised in Iringa and Njombe regions between October 2009 and December 2012; 59,597 males (54%) were circumcised at outreach sites and 50,516 males (46%) were circumcised at fixed sites (Figure 3). In Tanzania, males under the age of 25 represent 67.4% of the male population; males under the age of 25 accounted for 83.7% of males attending VMMC services in Iringa and Njombe.

Iringa and Njombe, Tanzania, introduced campaign-mode service delivery in 2010 using a combination of outreach and fixed sites. The number and frequency of campaigns increased subsequently and, in 2012, more circumcisions were performed in campaigns, predominantly during the winter season, than during routine services.

For all age groups, with the exception of those above the age of 35, clients attending campaign services were significantly younger than those attending routine services (p = 0.0001) in both campaign and routine service delivery combined. Majority of clients served during campaign (59%) were in the 10–14 years age group; The majority of clients served during routine service delivery (64%) were above 15 years of age (p<0.0001)

**Figure 4:**
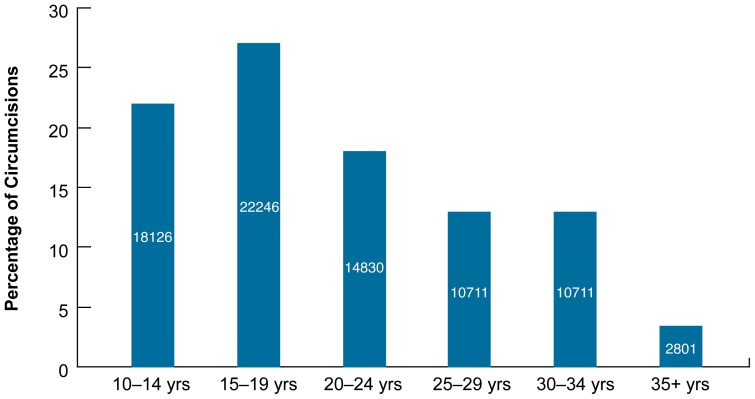
Percentage age distribution of VMMC clients in Zimbabwe, by 2009–2012.

**In Zimbabwe**, by December 2012, 82,391 men across all age groups (including infants which are not represented in Figure 4) had been circumcised since the start of the VMMC program in 2009. The estimated age distribution in Zimbabwe between 2009 to 2012 suggests that males under the age of 24 represent 64% of the male population.

**Figure 5:**
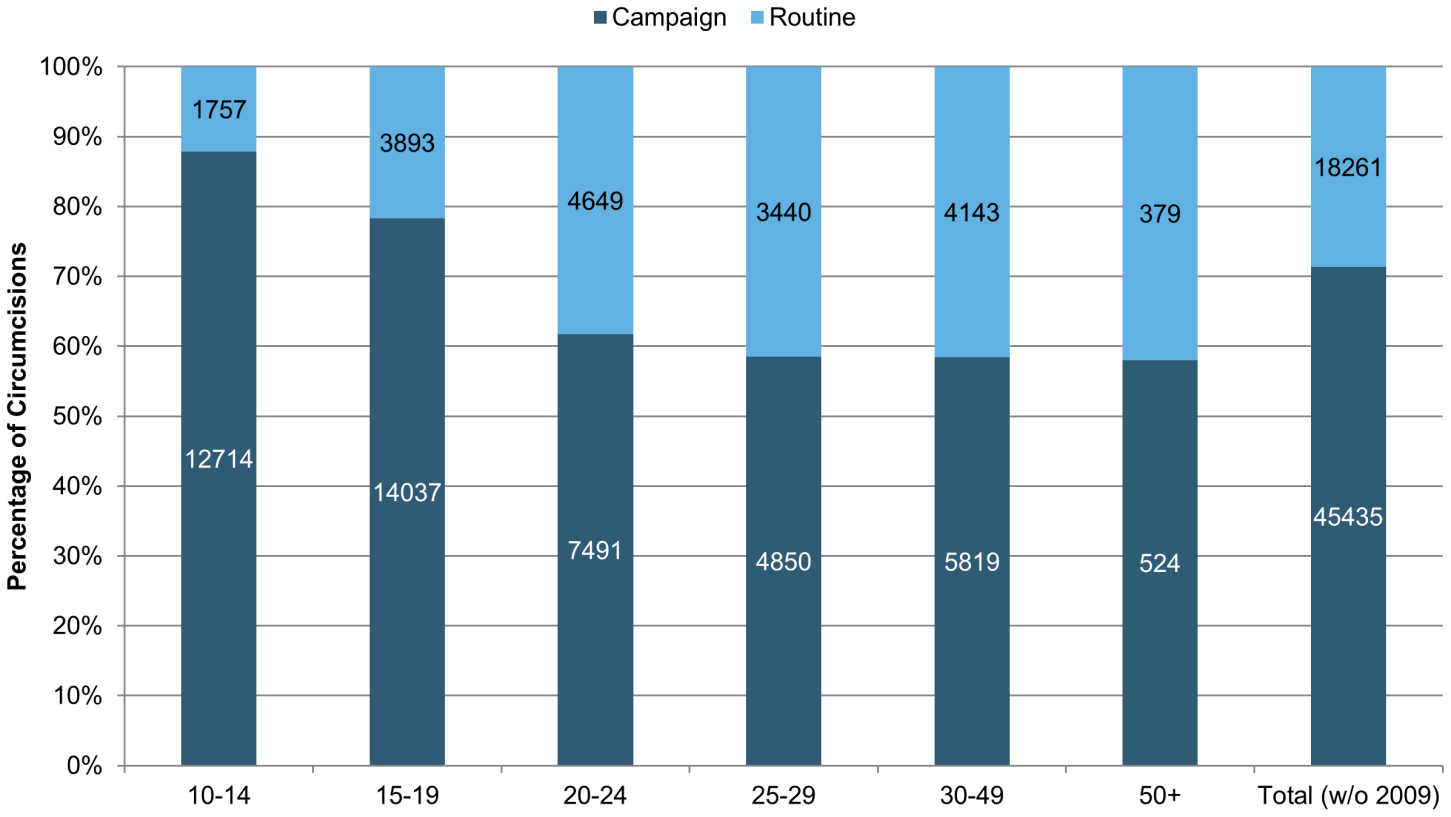
Percent distribution of circumcisions by intensity of service (campaign vs. routine), Zimbabwe, 2009–2012.

In Zimbabwe, significantly more male circumcisions were done during campaigns than during routine service delivery, 64% vs. 36% respectively (p<0.00001) (Figure 5).

During routine service delivery, the age distribution is more evenly distributed than in Tanzania through the various age categories and does include higher percentages of older age groups. Those aged 20–24 (48% campaign vs. 52% routine), 25–29 (45% campaign vs. 55% routine), 30–34 (45% campaign vs. 55% routine) and 50 and above (47% campaign vs. 53% routine) were reached almost evenly through campaign (mainly outreach) and routine (mainly fixed) service delivery.

The proportions of men from the age group 10–14, 15–19 and 20–24 accessing VMMC services during campaigns were significantly higher than those accessing routine services (p<0.05). However, for men above 25 years the differences between campaign and routine services are not significantly different (Figure 5).

## Discussion

**Figure 6:**
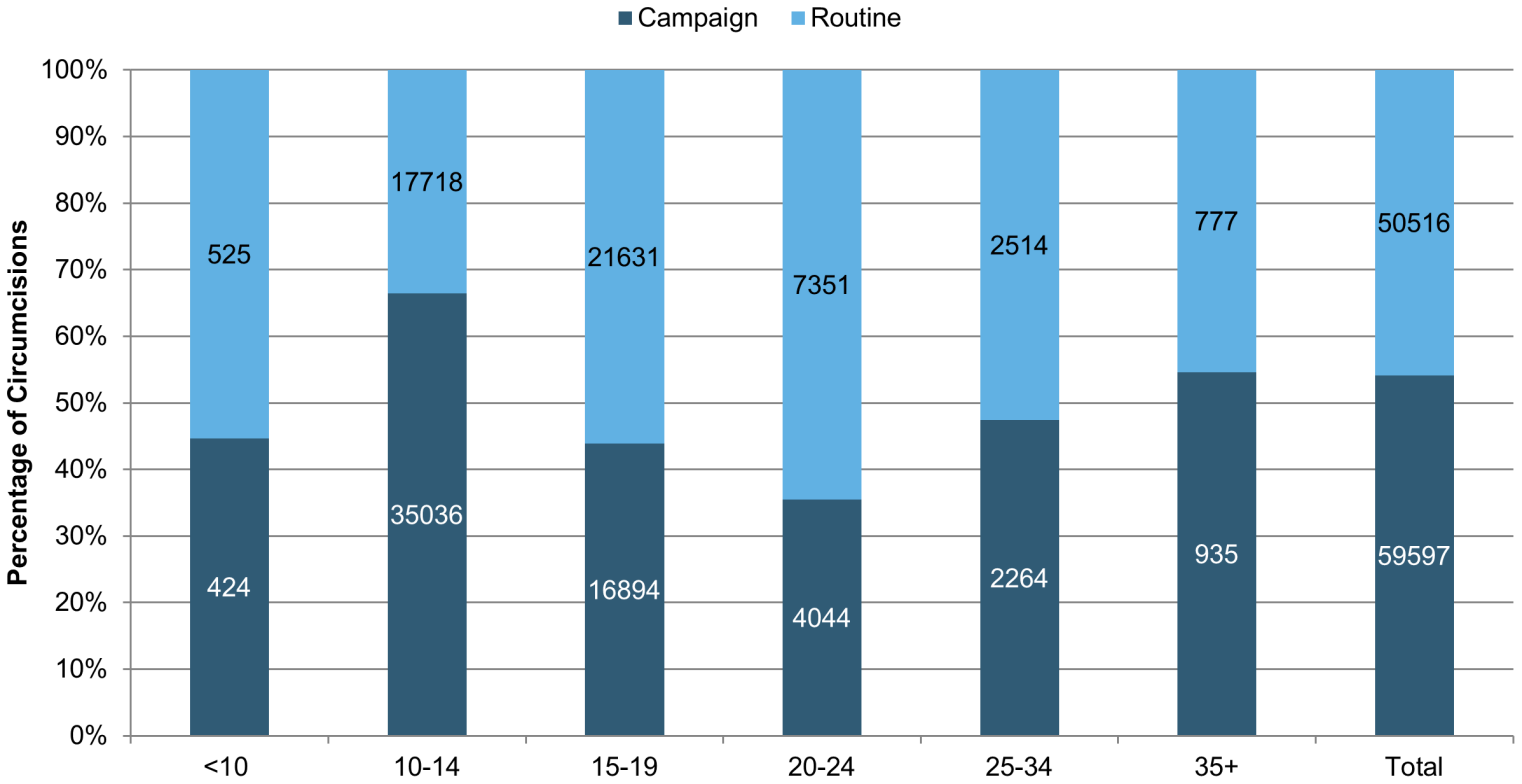
Percentage distribution of VMMC procedures by intensity (campaign vs. routine), in Iringa and Njombe regions, Tanzania, October 2009 to December 2012.

**In Tanzania**, the perception of respondents as well as program performance findings are consistent with formative research that was conducted in these regions (Iringa and Njombe), which indicates that older men do not come to receive VMMC services in a setting that includes predominantly adolescent clients. Moreover, there is already an underlying cultural perception that male circumcision is most appropriate before or during puberty, not in adulthood [11], which the respondents also discussed in this review. Campaign-based services, while increasing the number of circumcisions, seem to be an added barrier for older men; older men may avoid outreach services due to perceived lack of privacy in such high-volume settings. As shown above in Figure 6, men aged 20 and above do not utilize VMMC in similar numbers to younger men.

**In Zimbabwe**, cultural norms do not prescribe that circumcision should be done at a young age or during a specific season during the year, contrary to the case in Tanzania, respondents validated this belief. However, a higher proportion of younger clients access services during campaigns primarily because the campaigns are implemented during school holidays and campaign communications specifically target younger age groups and service delivery is facilitated at schools with support by head masters, teachers and parents. During routine service delivery outside of school campaigns, the program was able to attract older age groups above 25 years for VMMC. This is also supported by the finding that clients 25 years and above are not affected by the service delivery modality.

**In Tanzania**, the age-old wisdom that adolescence is the age of peer influence might be the reason for not only the increase in the number of circumcisions performed during campaign seasons but also for the disproportionate increase in the number of adolescents in such campaigns. Resistance to peer influence is more likely to increase between the ages of 14 and 18 than at earlier ages. There is little evidence for the increase in the ability to resist peer pressure between the ages of 10 and 14 [18]; therefore the client profile in Tanzania is consistent with the literature on adolescent psychosocial development in several respects. Thus, circumcision campaigns may feed on this peer pressure, in addition to the existing cultural preference for circumcision at a younger age. Conversely, older clients are not as affected by peer pressure as they are by social norms. On the other hand, once adolescents have turned their psychosocial attention toward matters of identity development, a shift that typically takes place late in adolescence and early adulthood [18], they may have developed the emotional wherewithal, as well as the need, to stand up to the influence of their peers. This may explain the situation in non-campaign routine service delivery approaches, where the majority of the clients are post-adolescent but the increase in utilization of services by this age group has not matched the increased utilization by adolescent clients during campaigns.

This is not the case **in Zimbabwe**, where an event such as a campaign fails to become a reason for peer pressure among adolescents only. Instead, both younger and older clients seek circumcision services when access is improved, either during campaign or non-campaign modalities. This signifies the importance of underlying cultural preferences—or the lack of preferences—on the response to programs that otherwise would attract a particular age group, as is the case in Tanzania. Zimbabwe is a country that does not traditionally circumcise men, contrary to Tanzania where most men are already circumcised and the prevalence of circumcision is higher than 80% in most regions [17].

The population age distribution figures (Figures 1 and 2) showed that the majority of the male populations in both Tanzania and Zimbabwe are under the age of 24. The difference in circumcision client profiles seen between Tanzania and Zimbabwe is difficult to attribute to one particular reason but highlights the variation in overall cultural and ethnic differences. To date, few research studies have examined socioeconomic or ethnic differences in resistance to peer influence, although there is some evidence that some communities may be more susceptible to peer pressure than others [18]. In general, ethnic and socioeconomic differences in adolescents' susceptibility to peer influence have received scant attention and might be one of the causes for the difference between Tanzania and Zimbabwe that needs further investigation.

Furthermore, the programs in Tanzania and Zimbabwe were at different stages in VMMC scale-up, with proportionally much fewer sites, either fixed or outreach, in Zimbabwe than in Tanzania. It is expected that with expansion of VMMC scale-up and wider access to service delivery outlets in Zimbabwe, the proportions of males reached during routine service delivery will increase compared to the proportion reached through campaigns and with it the representation of younger clients.

## Conclusion

Although service delivery modality and intensity affect the profile of clients coming for services, they do so within the underlying social context, which appears particularly powerful in Tanzania, and with the programmatic approach, which appears particularly influential in Zimbabwe. Therefore, the choice of service delivery needs to take into account such underlying differences. It is important to explore not only the service delivery modality and intensity but also the underlying cultural preferences and barriers in order to effectively target older as well as younger clients and scale up VMMC services.

While, it is crucial to continue exploratory efforts to increase service utilization by older client, it is, important to note that the adolescent population represents the largest proportion of the target population for the VMMC program in Tanzania and Zimbabwe. For example, in 2010, young people aged 15–24 accounted for 42% of new HIV infections in people aged 15–49 [19]. Among young people (15–24 years) living with HIV, nearly 80% (4 million) live in sub-Saharan Africa. VMMC intervention for the adolescent age group (10–19 years) and youth (20–24 years) should be worthwhile in all priority countries in Southern and Eastern Africa [19]. VMMC can be a broad platform for both younger and older clients in the fight against HIV. Formative research should be an integral part of VMMC programming to address socio-cultural barriers and to design service delivery modalities that meet specific needs and desires of communities as well as specific age groups of men.

## Limitations

The qualitative part of this review looked at only perspectives of those involved in the supply side of VMMC services and not that of clients.
